# Antifungal Properties of Chemically Defined Propolis from Various Geographical Regions

**DOI:** 10.3390/microorganisms10020364

**Published:** 2022-02-04

**Authors:** Marcin Ożarowski, Tomasz M. Karpiński, Rahat Alam, Małgorzata Łochyńska

**Affiliations:** 1 Department of Biotechnology, Institute of Natural Fibres and Medicinal Plants, Wojska Polskiego 71b, 60-630 Poznań, Poland; marcin.ozarowski@iwnirz.pl; 2 Chair and Department of Medical Microbiology, Poznań University of Medical Sciences, Wieniawskiego 3, 61-712 Poznań, Poland; 3 Department of Genetic Engineering and Biotechnology, Faculty of Biological Science, Jashore University of Science and Technology, Jashore 7408, Bangladesh; rahatalam1643@gmail.com; 4 Laboratory of Computational Biology, Biological Solution Centre (BioSol Centre), Jashore 7408, Bangladesh; 5 Department of Bioeconomy, Institute of Natural Fibres and Medicinal Plants, Wojska Polskiego 71b, 60-630 Poznań, Poland; malgorzata.lochynska@iwnirz.pl

**Keywords:** propolis, fungal strains, *Candida*, extracts, phenolic compounds

## Abstract

Long-term fungal infections that are difficult to treat require new substances for their prevention, treatment, or as adjuvants during antibiotic therapy. Propolis is a very promising source of natural substances that show a wide range of pharmacological properties, including antifungal activity against various fungal strains. The purpose of the literature review was to summarize recent studies (PubMed, Scopus) on progress in evaluating the antifungal activity of chemically defined propolis extracts. During the selection of studies, only those with results of antifungal activity expressed as minimal inhibitory concentration (MIC) and/or minimal fungicidal concentration (MFC) were analyzed. Moreover, plant, animal and environmental factors influencing the chemical composition of propolis are discussed. Mechanisms of antifungal activity of propolis extracts and research trends in the aspect of developing new therapies and the assessment of drug interactions are indicated. The review of the research results shows that there is great progress in the definition of propolis extracts. After comparing the MIC/MFC values, it was assessed that propolis extracts offer a wide range of activity not only against pathogenic *Candida* strains but also against risky molds; however, the strength of this activity is varied.

## 1. Introduction

Propolis (common names: bee glue, hive dross) is defined as a natural resinous mixture which is produced by honey bees (*Apis mellifera*) using secretions collected from trees and herbaceous plants or resins, mucilage, gums from the flowers, fruits, branches, stem, and leaves of different plants [1,2,3,4]. Moreover, in detail, propolis is composed of the following sources: (1) resins from plants which are collected by *Apis* *mellifera*, (2) wax from the metabolism of bees, (3) substances added by *Apis* *mellifera* when making propolis [1], and (4) other components such as essential oils and pollen [2]. Nainu et al. [5] described a more detailed main composition of raw propolis with the percentage range of components: resins and balms (from 50% to 60%), waxes and fatty acids (from 30% to 40%), essential oils (5–10%), and other chemical compounds (to 5%), mainly enzymes (i.e., glucose-6-phosphatase; adenosine triphosphatase; acid phosphatase), vitamins and minerals.

Propolis has been known and used as a medicinal product since the ancient Greeks, Romans and Egyptians until the present day [6]. The complex composition of propolis makes that natural product exert a broad spectrum of pharmacological activities. One of the scientific problems that has received a lot of attention is the origin of propolis and the influence of geographic and environmental factors on its chemical composition [1,3,7] and also on pharmacological activities [8,9,10].

Apart from other bee products, such as honey, royal jelly and bee venom, propolis is also a significant source of natural chemical compounds [11,12]. Currently, numerous studies have been carried out on propolis in various fields. With the advancement of research methods and scientific curiosity, the number of results about propolis continue to increase. This continuing trend is consistent with the need to use products of natural origin, not only in treatment but also in the prevention of numerous diseases.

Propolis has shown mainly antifungal [8,9,13,14,15,16,17,18,19,20,21,22], antibacterial [10,19,20,23,24], antiviral [20,25,26,27,28,29], antiparasitic [5,20,30,31], anti-inflammatory [17,32,33,34] and neuroprotective activity [35,36,37,38], and also anticancer properties [39,40,41,42].

**Figure 1:**
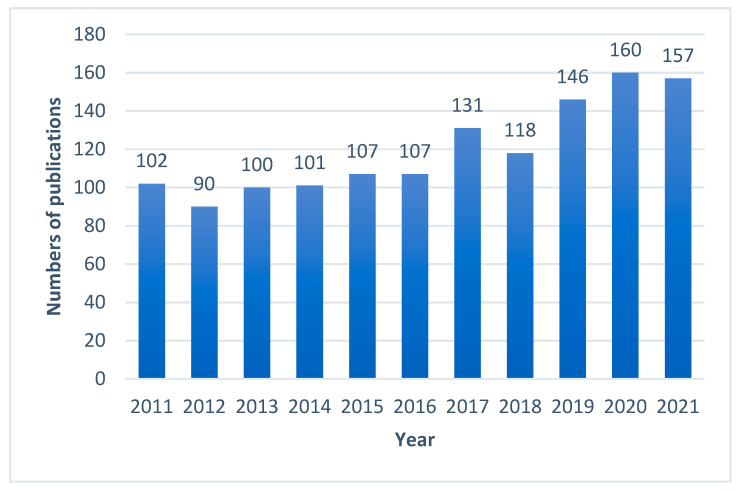
Numbers of scientific outputs containing the words “antimicrobial activity” and “propolis” as articles in all categories available in the PubMed database for the last 10 years (2011–2021).

**Figure 2:**
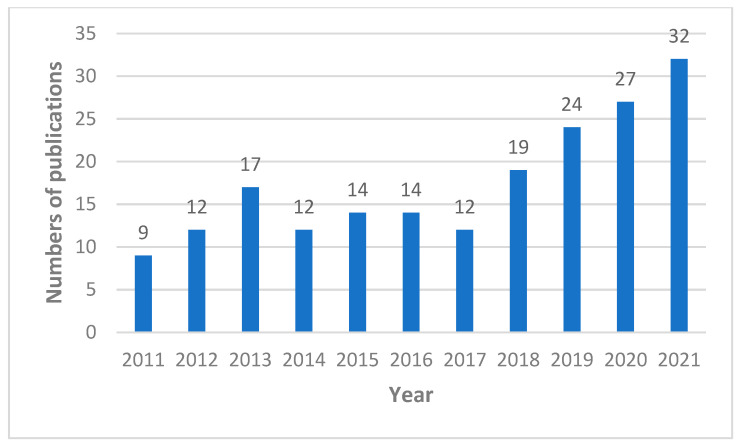
Numbers of scientific outputs containing the words “antifungal” and “propolis” as articles in all categories available in the PubMed database for the last 10 years (2011–2021).

**Figure 3:**
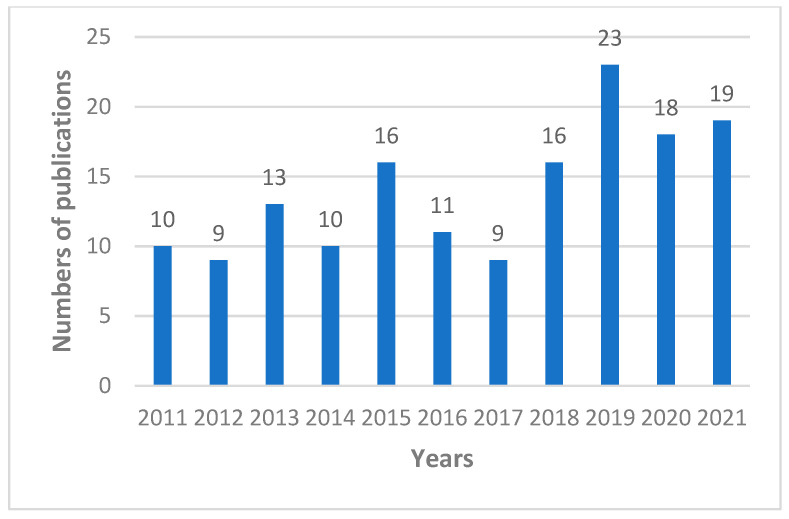
Numbers of scientific outputs containing the words “*Candida*” and “propolis” as articles in all categories available in the PubMed database for the last 10 years (2011–2021).

In the largest medical bibliographical database PubMed, 3885 publications about propolis are available, however there are 2447 publications from the last 10 years (2011–2022). The number of scientific articles is growing i.e., in 2021 423 were published, in 2020 375, and in 2019 322. Taking into account the research topic, it can be summarized that in the last 10 years as many as 1072 publications on antimicrobial activity of propolis have been published (Figure 1), including 161 publications about antifungal properties of propolis (Figure 2), 122 articles about “*Candida*” and “propolis” (Figure 3) and 26 articles about “propolis” and “candidiasis” (as keywords). Among these publications, only two results of clinical trials have been published. At this point, it should be emphasized that the PubMed database includes 1737 scientific articles published in the years 1975–2022. This gives rise to a review of published research results in this area.

## 2. Plant, Animal and Environmental Factors Influencing the Chemical Composition of Propolis

It is well known that many factors may influence the chemical composition of propolis such as plant sources, geographical region and climatic conditions, time of production of propolis and type of *Apis mellifera* [1,5,7,43].

**Figure 4:**
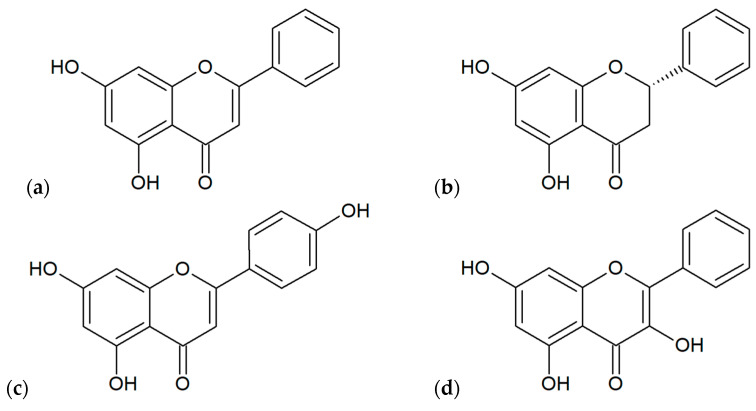

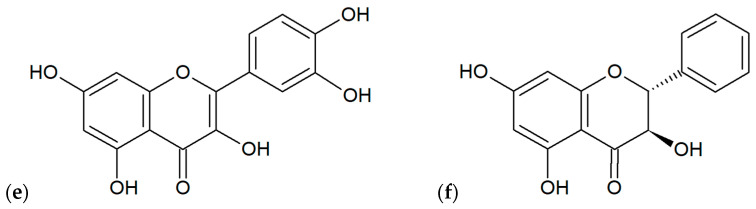
Main flavonoids found out in various extracts of propolis: (**a**) chrysin, (**b**) pinocembrin, (**c**) apigenin, (**d**) galangin, (**e**) quercetin, (**f**) pinobanksin.

**Figure 5:**
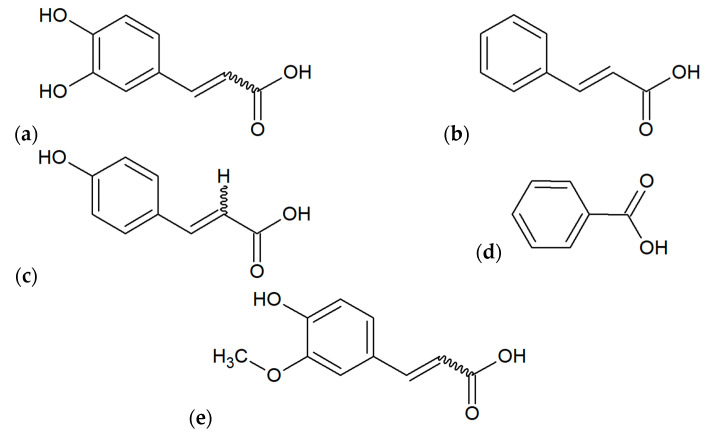
Main phenolic acids found out in various extracts of propolis: (**a**) caffeic acid, (**b**) cinnamic acid, (**c**) p-coumaric acid, (**d**) benzoic acid, (**e**) ferulic acid.

The most popular plant species as a source of biomaterial for the production of propolis include: *Acacia spp.*, *Aesculus hippocastanum*, *Betula pendula*, *Fagus* sp., *Fraxinus* sp., *Pinus* spp., *Prunus* spp., *Quercus* sp., *Salix alba* [1]. In Poland, very important plant sources are *Alnus* spp. and *Populus* spp. [10]. Plant materials obtained from mentioned species are chemically different because these plants contain various primary and secondary metabolites classified mainly to phenolic compounds (flavonoids, phenolic acids, terpenoids; Figure 4 and Figure 5) and essential oils [1]. In addition to this, the situation is more complicated because the chemical composition of finished products obtained from propolis may depend on the physicochemical processes used during the extraction (e.g., solvent, extraction temperature and time, standardization), similarly as during processing using other plant materials [44]. However, Pobiega et al. [43] did not observe differences in the qualitative composition of propolis extracts obtained by various methods such as traditional extraction with 70% ethanol (1:10 and 1:5 propolis to ethanol during 1–7 days) and shaking and ultrasound-assisted shaking extractions.

Moreover, the chemical diversity of propolis is dependent on the bee species in different regions of the world [2]. The main producer of propolis is believed to be *Apis mellifera*, which is the most common species of honey bee in Europe, Africa, and the Middle East. However, the honey bee genus *Apis* consists of 11 species [45]. Furthermore, in tropical and subtropical regions of Africa, Asia and America are also well-known stingless honey bees (or meliponines) i.e., in Africa—African stingless bee—*Meliponula ferruginea* [46,47]. This type of propolis is traditionally used in medicine in various regions of Argentina, Brazil Mexico, and Vietnam [46]. It has been asserted that the Caucasian honey bee (*Apis mellifera* caucasia) is able to produce between 250 and 1000 g of propolis annually, per hive [2].

Increasingly, propolis is classified as honey bee propolis and stingless bee propolis [2]. Actually, 502 different chemical compounds in propolis obtained by honey bees have been described, and more than 100 chemical compounds produced by stingless bees [2]. According to the analysis of Tran et al. [2] phenolic compounds are dominant in honey bee propolis (79.5%) and also in stingless bee propolis (63.0%). The second group of chemical compounds are the terpenoids accounted for 18.9% (honey bee propolis) and 37.0% (stingless bee propolis) of all compounds found in propolis. However, the chemical diversity of propolis composition is still extensively studied.

The qualitative and quantitative chemical composition of propolis differs in various geographical regions of the world. Recently, a few studies have been carried out about i.e., African propolis [48], Anatolian propolis [49], Brazilian red propolis [8,40], Brazilian green propolis [35], Brazilian organic propolis [17], Egyptian propolis [37], Indian propolis [38], Korean propolis [32], Lebanese propolis [33], Nepalese propolis [50], propolis from Turkey [41], and Taiwanese green propolis [24]. Among various propolis, the Brazilian propolis is commonly classified into 13 types based on their properties (mainly: color, chemical composition, texture), and on geographic origin [40,51,52]. Other authors mentioned the main types of propolis such as Brazilian and European propolis [53].

In Table 1 is presented the comparison of chemical compositions of propolis extracts, which have been currently estimated for antifungal properties. Generally, ethanolic extracts of propolis, which come from different regions of Brazil, are investigated most often (199 publications in PubMed between 2011 and 2021) [8,14,17,40,54]. High-quality chemical analysis mainly concerns two types of propolis such as Brazilian red propolis (using gas chromatography coupled to mass spectrometry [54], and liquid chromatography–high-resolution mass spectrometry [53]), and Brazilian green propolis (using liquid chromatography–high-resolution mass spectrometry [53], and high-performance liquid chromatography (HPLC-DAD) [13].

## 3. Antifungal Properties of Propolis

Antifungal activities of various extracts of propolis have been examined against several yeasts, such as *Candida albicans*, *C. dubliniensis*, *C. glabrata*, *C. krusei*, *C. parapsisolis*, *C. tropicalis*, *Saccharomyces cerevisiae* [13,16,17,18,21,50,54,55,56,57,60,61,62,63,64], as well as against molds, such as *Alternaria solani*, *Alternaria alternata*, *Aspergillus niger*, *Aspergillus ochraceus*, *Botrytis cinerea*, *Cladosporium* spp., *Fusarium solani*, *Fusarium oxysporum*, *Mucor mucedo*, *Penicillium digitatum*, *Penicillium expansum*, *Penicillium chrysogenum*, *Rhizopus stolonifera*, *Rhodotorula mucilaginosa* and *Trichophyton* spp. [9,19,43,64].

Furthermore, there is an increasing amount of scientific evidence on the mechanism of antifungal activity after the use of propolis extracts. Corrêa et al. [65] and Gucwa [57] showed that the cell membrane of fungi may be a possible target of an extract of propolis besides the induction of cell death. Earlier, researchers showed that an extract of propolis can inhibit the activity of extracellular phospholipase, leading to attenuation of the fungal cell adhesion to epithelium [66]. Currently, it has been observed that propolis may influence the formation and integrity of the cell wall of fungi and can inhibit the morphological transformation of *C. albicans* [57]. Stahli et al. [53] observed that an ethanolic extract of propolis caused a loss of the cell wall integrity of *C. albicans* and decreased the metabolic activity. Okińczyc et al. [50] showed that ethanolic extract of propolis inhibited filamentation of cells of *C. albicans,* germination of yeasts and increased production of the superoxide anion radical. A few studies showed that extracts of propolis are effective inhibitors of biofilm [16,53,54,57,67,68,69]. Freires et al. [54] observed that ethanolic extract of Brazilian propolis (types 3 and 13) can lead to disruption of the biofilm structures of *Candida* sp. at concentrations <0.9 μg/mL. Moreover, Martorano-Fernandes et al. [67] revealed that extract from Brazilian red propolis possessed inhibitory effects on the proliferation and diminished the metabolism of biofilms of *C. albicans*. Fernández-Calderón et al. [16] showed that 70% ethanolic extract of Spanish propolis inhibited the fungal biofilm at sub-inhibitory concentrations of extract (0.1 and 0.05%). Gucwa et al. [57] showed that values of minimum biofilm eradication concentration of Polish propolis extract were in the range of 0.04% to more than 1.25% (*v*/*v*) for *C. glabrata*, *C. krusei*, and *C. albicans*. Gucwa obtained also very interesting results about a synergistic effect of extract of Polish propolis with voriconazole, and fluconazole against *C. albicans* [57]. Pharmacological aspects of interactions have been investigated also by Argüelles [70], who showed that combination of propolis with carnosic acid (diterpene occurs in *Rosmarinus officinalis* and *Salvia officinalis*), through synergistic action, may lead to a drastic reduction in survival of *Candida albicans* cells, leading to a fungicidal effect. These observations open a new scientific window for the application of this combination in clinical therapies of *C. albicans* infections.

Therapy of various candidiasis should be based on complementary methods, including natural products such as not only tea tree and garlic but also propolis [71]. It should be emphasized that the number of studies on the evaluation of the effects of propolis in vulvovaginal candidiasis (opportunistic fungal infection) is increasing [25,27,71,72]. New trends in antifungal research of propolis include also assessment of anti-candidal activities i.e., in orthodontic materials (green propolis) [73], dental surface (red propolis) [61,74], in treatment of chronic periodontitis (red propolis) [75], in caries (Brazilian and European propolis) [53]. The third research area is endemic and rare diseases. Santos et al. [14] showed that Brazilian red propolis exerted antifungal activity against *Paracoccidioides brasiliensis*. This pathogenic fungal strain can cause systemic mycosis (paracoccidioidomycosis), also known as South American blastomycosis, classified as a neglected tropical disease [76]. In all these cases, extracts of propolis showed antifungal activities (Table 2). In addition, the results of these studies indicate an interesting application of propolis extracts in vulvovaginal candidiasis, dental diseases and fungal tropical diseases.

## 4. Collected Data Analysis

**Figure 6:**
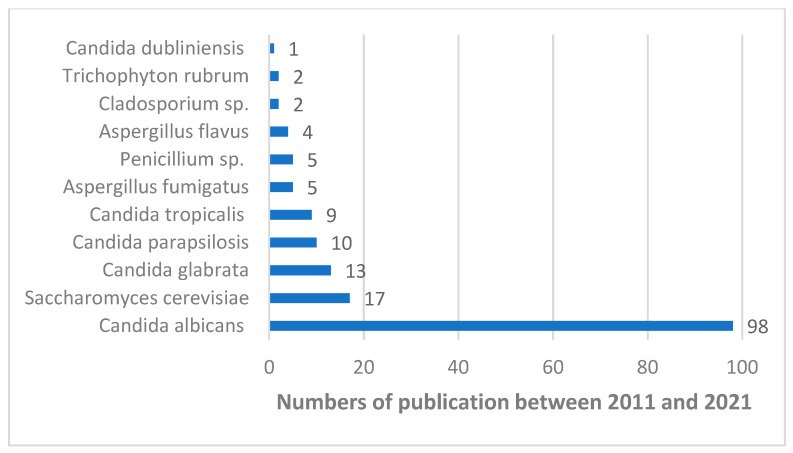
A number of publications available in the PubMed database with the ten most-analyzed fungal strains in terms of sensitivity to propolis extracts (at last ten years).

In recent years, the authors have used new standard methods for the determination of minimal inhibitory concentration (MIC), and minimal fungicidal concentration (MFC) for chemically defined extracts from propolis, which are key parameters for this review (Table 2). In more than half of the publications, the chemical compositions of extracts have been characterized using HPLC methods, which have been shown in Table 1. In other cases, reference was made to foreign results of phytochemical tests. The chemical composition and origin of propolis were very diverse and this has an impact on the strength of the antifungal activities of propolis extracts. The antifungal activities of propolis extracts are most frequently tested in vitro on *Candida albicans* in comparison with *C. dubliniensis*, *C. glabrata*, *C. krusei*, *C. tropicalis*, *C. parapsilosis*, *Saccharomyces cerevisiae* and strains of molds. The most popular species of pathogenic fungi tested in the last 10 years in microbiological studies are shown in Figure 6.

After comparing the results of studies assessing the antifungal activity of different propolis, it can concluded that all tested extracts of propolis inhibited *Candida* sp. However, most of the studies on the antimicrobial effect of propolis do not inform about the source of clinical strains of pathogenic fungi. It was shown that the most active were the following ethanolic extracts: Irish propolis (MIC = 0.3 μg/mL) [21] > Czech propolis (MIC = 0.6 μg/mL) [21] > Brazilian reed propolis (MIC = 2.0 μg/mL) [54] > Brazilian poplar propolis (MIC = 4 μg/mL) [54] > German propolis (MIC = 5 μg/mL) [21] > Iranian propolis (MIC_50_ = 21 μg/mL) [63] > French poplar propolis (MIC = 31.25 μg/mL) [56] > Portuguese propolis (MIC_50_ = 32.0 μg/mL) [64] > Brazilian reed propolis (MIC = 32–64 μg/mL) [75] > Brazilian organic propolis (MIC = 100 μg/mL) [17] > Cameroonian propolis (MIC = 125–500 μg/mL) [18] > Nepalese propolis (MIC = 256 μg/mL) > Brazilian green propolis (MIC = 2–4 mg/mL) [13] > Polish propolis (MIC = 2–32 mg/mL) [43]. Moreover, in five studies [13,17,18,54,75] antifungal activities of extracts of propolis against three or more species of *Candida* were compared. Many authors demonstrated that *C. albicans* is more sensitive than other species of *Candida* (C. *dubliniensis*, *C. glabrata*, *C. krusei*, *C. tropicalis*, *C. parapsilosis*) to ethanolic extracts from various propolis.

Often the authors described that phenolic compounds are the main components which can be responsible for the antifungal activities of propolis extracts.

There is increasing information that not only polyphenolic compounds, but also essential oil components found in propolis exhibit antimicrobial activity [Ikeda, 2021. It was shown that essential oils from Brazilian propolis contain i.e., camphene, p-cymene, limonene, myrcene, α-pinene, β-pinene [55]. Many studies have proven that these chemical components occurring in essential oils of medicinal plants exerted antifungal activity mainly against *Candida albicans* [60,77,78,79]. After comparison of phytochemical components of propolis extracts (Table 1) it is very difficult to conclude which chemical compounds are the most active against fungal strains. Therefore, it can be concluded that the synergy of action of all components of propolis extract determines the antifungal activity. However, it should be noted that the extracts of Brazilian propolis showed very wide variability in antifungal activity, e.g., according to Freires et al. [54] Brazilian reed propolis showed MIC = 2.0 μg/mL), Siqueira et al. [75] revealed for the same type of propolis showed MIC = 32–64 μg/mL, whereas Sokolonski et al. [13] stated values of MIC from 0.5 to more than 8 mg/mL (Table 2). This indicates the need to standardize the chemical composition of the different types of propolis collected all over the world.

## 5. Conclusions

First, based on the review of the bibliography, it was found that in recent years studies have focused mainly on the assessment of the susceptibility of *Candida albicans* strains to ethanolic extracts of various propolis.

Second, these extracts often contain different chemical compositions, resulting from the unique flora of the region where bees produce propolis. The wide range of demonstrated activity against *Candida albicans* leads to the conclusion that the need to standardize the chemical composition of propolis extracts should be considered, especially in the case of the same types of propolis.

Third, during in vitro studies, the isolated strains of pathogenic fungi should be more accurately described in terms of patients’ age and gender, their diseases, and different treatments. These factors may influence the sensitivity of clinical strains to the action of propolis extracts.

Fourth, chemical, microbiological and pharmacological tests of chemically defined propolis extracts should be performed for a wider group of pathogenic fungi.

Fifth, the antifungal activity of propolis should be confirmed in clinical trials, not only in terms of efficacy and safety assessment but also in order to demonstrate pharmacological interactions with other drugs.

## Figures and Tables

**Table 1:** Examples of the chemical composition of propolis produced by *Apis mellifera* from various geographical regions using to an evaluation of pharmacological studies.

Name of Propolis	Plant Source	Extract	Main Chemical Classes of Phytocomponds	Ref.
Brazilian red propolis Brazilian green propolis	*Dalbergia ecastophyllum*	Ethanolic extract	identical qualitative composition: chlorogenic acid, 4-hydroxycinnamic acid, luteolin 7-rutinoside, baccharin, aromadendrin 4′-methylether 7-rhamnoside, 3,4-dicaffeoylquinic acid, 3,5-dicaffeoylquinic acid, 1,5-dicaffeoylquinic acid, kaempferol 7-methylether 4′-glucoside, di-caffeoyl quinic acid, quercetin, luteolin 5-methyl ether, pinocembrin, drupanin, viscidone, chrysin, pinocembrin-5-methyl ether, benzyl caffeate, kaempferol-7-methyl ether, 4-hydroxycinnamic acid, luteolin 7-rutinoside, baccharin	[53]
Brazilian red propolis	*Dalbergia ecastophyllum*	Ethanolic extract	38 phenolic compounds including 26 without identification: calycosin, luteolin, (3S)- vestitol, (3S)-neovestitol, medicarpin, pterocarpan, isoliquiritigenin, liquiritigenin, biochanin A, retusapurpurin A, formononetin,	[8]
Brazilian propolis type 3 (southern Brazil) type 13 (Maceio City, Alagoas State, northeastern Brazil)	Type 3—poplar propolis—from *Populus* L. trees, Salicaceae family)Type 13- red propolis—from *Dalbergia ecastophyllum*	80% ethanolic extract	p-coumaric acid, caffeic acid phenethyl ester, kaempferol, quercetin, medicarpin, vestitol, formononetin	[54]
Brazilian red propolis from the city of Santo Antônio; type 13	*Dalbergia ecastophyllum*	Hexane extract	saturated and unsaturated aromatic hydrocarbons, ketones (i.e., 2 (3H)—furanone), alcohols (i.e., triacontanol), ethers (Methyleugenol, Isopropyl tetracosyl ether), terpenes (Lupenone, Lupeol, Lupeol acetate)	[40]
Brazilian red propolis from the Maceio city, Alagoas State, Northeast of Brazil; type 13	*Dalbergia ecastophyllum*	80% ethanolic extract	biochanin A, daidzein, formononetin, isoliquiritigenin, liquiritigenin, neovestitol, quercetin, vestitol	[14]
Brazilian organic propolis from the city of Palmital	No information	Ethanolic extract	quercetin, 3,4-dicaffeoylquinic acid, trans-caffeoyltartaric acid, caffeoyltartaric acid, gibberelins A7, A9, A20	[17]
Brazilian crude organic propolis	No information	Essential olis from crude propolis (after hydrodistilation)	14 chemical compounds i.e., camphene, p-cymene, limonene, myrcene, α-pinene, β-pinene, sabinene, thuja-2,4(10)-diene, tricyclene	[55]
Czech propolis	No information	Ethanolic extract	Main phenyl carboxylic acids such as: caffeic acid, cinnamic acid, p-coumaric acid, benzoic acid Main flavonoids such as: chrysin, galangin, pinocembrin, 2,3-dihydrobenzofuran	[21]
French poplar-type propolis	*Populus* species	Dichloromethane extract	49 chemical composition; mainly: pinobanksin-3-acetate, pinocembrin, chrysin, galangin, prenyl caffeate, 2-acetyl-1,3-dicoumaroylglycerol, caffeic acid, *p*-coumaric acid, ferulic acid, isoferulic acid, 3,4-dimethoxycinnamic acid, 4-methoxycinnamic acid, pinobanksin, naringenin	[56]
Germany propolis	No information	Ethanolic extract	Main phenolic acids such as: benzoic acid, cinnamic acid, 4-methoxyphenyl propanoic acid, dodecanoic acid, myristic acid, salicylic acid, hexadecanoic acid, 4-vinyl-2-methoxy-phenol, 2,3-dihydrobenzofuran	[21]
Irish propolis	No information	Ethanolic extract	Main flavonoids such as: chrysin, galangin, pinocembrin and significant amounts of caffeic acid, nonacosane, pentacosane, heptacosane, eudesmol, guaiol, and alpha-bisabolol, and 2,3-dihydrobenzofuran	[21]
Indian propolis from Bharatpur, Rajasthan, India	No information	Ethanolic extract	caffeic acid phenethyl ester, galangin	[38]
Nepalese propolis from the Korak village (Nepal)	*Dalbergia* sp.	Ethanolic extract	23 chemical compounds, mainly: butein, butin, cearoin, dalbergin, 6,7-dihydroxyflavanon, 2’,7-dihydroxy-5-methoxyisoflawan, formononetin, 2’-hydroxyformononetin, isoliquiritigenin, liquiritigenin, medicarpin, 4-metoxydalbergione, 4’-methoxy-2’,3, 2-(1-phenylprop-2-enyl)benzene-1,4-diol, obtusaquinol, pinocembrin, plathymenin, retusapurpurin B or A isomer 7-trihydroxyisoflavanone	[50]
Polish propolis collected from the following Voivodeships: Greater Poland, Lesser Poland, Masovian, Łódź, Podlaskie, Warmian-Masurian	No information	70% Ethanolic extract	27 phenolic compounds, mainly: acacetin; apigenin; apiin; caffeic acid; (+)-catechin; chrysin; cichoric acid; cinnamic acid; cinnamyl alcohol; p-coumaric acid; dimethyl caffeic acid; ellagic acid dehydrate; ferulic acid; galangin; 3:4- hydroxybenzoic acid; isorhamnetin; kaempferol; 4-methoxycinnamic acid; oroxylin; pinobanksin; pinocembrin; (+/−)-pinostrobin; protocatechuic acid; quercetin; quercetin-3-O-rutinoside; syringic acid; vanillic acid; Main flavonoids: pinocembrin, 5,7-dihydroxyflavone (chrysin), pinobanksin, apigenin, kaempferol Main phenolic acids: 4-hydroxycinnamic acid (p-coumaric acid), 4-hydroxy-3-methoxycinnamic acid (ferulic acid), and 3,4-dihydroxycinnamic acid (caffeic acid)	[43]
Polish propolis collected from the Warmia-Masuria Province in northeastern Poland	No information	70% and 96% ethanolic extracts	Flavonoids: apigenin, chrysin, galangin, kaempferol, naringenin pinobanksin, pinocembrin, quercetinPhenolic acids: caffeic acid, coumaric acid, ferulic acid, syringic acid, vanillic acid, cinnamic acid, p-Hydroxybenzoic acid	[9]
Polish propolis collected from northeastern Poland	No information	ethanolic extracts	43 chemical compounds, i.e., Phenolic acids: Hydroxybenzoic acid, Caffeic acid, p-Coumaric acid, Ferrulic acid, Flavonoids: Galangin, Acacetin, Quercetin, Kaempferol, Apigenin, Naringenin, Pinobanksin-3-O-acetat, Pinobanksin-3-O-propionate, Pinobanksin-3-O-butyrate, Pinobanksin-3-O-penatnoate	[57]
Polish propolis	*Populus nigra*	dichloromethane extract	85 chemical compounds including: 13 aromatic acids i.e., benzoic acid, p-coumaric acid, dimethoxycinnamic acid, ferulic acid 5 other aromatics i.e., benzyl alcohol, vanilin 3 fatty acids i.e., linoleic acid, oleic acid, palmitic acid 28 esters i.e., cinnamyl p-coumarate, benzyl p-coumarate, pentyl p-coumarate, pentenyl p-coumarate, 29 flavonoids and chalcones i.e., 3-acetylpinobanksin, alpinon, alpinon chalcone, chrysin, galangin, isosakuranetin, isosakuranetin chalcone, kaempferol-methyl-ether, pinobanksin, pinocembrin, pinocembrin chalcone 2 other compounds	[58]
Thailand propolis, commercially available	No information	ethanolic extract	gallic acid, quercetin, pinocembrin, chrysin, and galangin	[59]
Taiwanese green propolis (collected from different regions in Taiwan)	No information	95% ethanolic extract	Prenylated flavanone derivatives: Propolin C, D, F, G Sum of propolins = 37.55 ± 1.29 (in 95% ethanolic extract)	[24]
Turkish propolis	*Populus nigra*	ethanolic extract	3-Omethylquercetin, chrysin, caffeic acid, caffeic acid phenethyl ester, galangin, pinocembrin	[41]

**Table 2:** The activity of different propolis’ extracts against fungal strains estimated by MIC—minimal inhibitory concentration, MFC—minimal fungicidal concentration.

Preparation	Fungal Strains	Results		Ref.
		MIC (μg/mL)	MFC (μg/mL)	
Brazilian propolis, type 3 of propolis, ethanolic extract (chemical characteristic in Table 1)	*Candida albicans* (CBS 562)	4	>500	[54]
	*Candida dubliniensis* (CBS 7987)	4		
	*Candida glabrata* (CBS 07)	7.8	250	
	*Candida krusei* (CBS 573)	15.6	500	
	*Candida tropicalis* (CBS 94)	31.3	>500	
	*Candida parapsilosis* (CBS 604)	31.3	>500	
Brazilian propolis, type 13 of propolis, ethanolic extract (chemical characteristic in Table 1)	*C. albicans* (CBS 562)	2.0	250	[54]
	*C. dubliniensis* (CBS 7987)	1.0	250	
	*C. glabrata* (CBS 07)	7.8	250	
	*C. krusei* (CBS 573)	4	500	
	*C. tropicalis* (CBS 94)	4	250	
	*C. parapsilosis* (CBS 604)	2	500	
Brazilian organic propolis (chemical characteristic in Table 1)	*C. albicans* (MYA 2876)	100	200	[17]
	*C. glabrata* (ATCC 90030)	100	200	
	*C. tropicalis* (ATCC 750)	200	400	
	*C. krusei* (ATCC 6258)	50	100	
	*C. parapsisolis* (ATCC 22019)	100	200	
Brazilian green propolis –ethanolic extract	*C. albicans* (2508, 2517, 3703, 3704)	2–4 mg/mL	4–8 mg/mL	[13]
	*C. albicans* (PAC: 06, 08, 13, 15, 17–20)	>8 mg/mL	>8 mg/mL	
	*C. dubliniensis* (PAC 01)	2 mg/mL	>8 mg/mL	
	*C. tropical* is (PAC: 02, 04, 05, 15)	1–4 mg/mL and > 8	>8–4 mg/mL	
Brazilian red propolis –ethanolic extract	*C. albicans* (2508, 2517, 3703, 3704)	0.5–1 mg/mL	1–4 mg/mL	
	*C. albicans* (PAC: 06, 08, 13, 15, 17–20)	>8–1 mg/mL	>8–1 mg/mL	
	*C. dubliniensis* (PAC 01)	1 mg/mL	2 mg/mL	
	*C. tropicalis* (PAC: 02, 04, 05, 15)	0.125–1 mg/mL	1–4 mg/mL	
Brazilian red propolis –hydroalcoholic extract	*C. albicans* ATCC 90028	0.29 mg/mL	1.17 mg/mL	[61]
Brazilian red propolis –ethanolic extract	*C. albicans*—12 clinical isolated strains	32–64	64–512	[75]
	*C. albicans*—12 clinical isolated strains	MIC_50_ = 32		
	*C. albicans*—12 clinical isolated strains	MIC_100_ = 64		
	*C. tropicalis*—5 clinical isolated strains	64	64–256	
	*C. tropicalis*—5 clinical isolated strains	MIC_50_ = 64		
	*C. tropicalis*—5 clinical isolated strains	MIC_100_ = 64		
	*C. glabrata*—2 clinical isolated strains	32–64	64	
	*C. glabrata*—2 clinical isolated strains	MIC_50_ = 64		
	*C. glabrata*—2 clinical isolated strains	MIC_100_ = 64		
Brazilian red propolis (chemical characteristic in Table 1)	*Paracoccidioides brasiliensis* (Pb18) strain	reduction the viability of the fungal strain in 75% (at 24 h), up to 92% (at 72 h) after concentration at 500 mg/mL		[14]
Brazilian red propolis –ethanolic extracts (chemical characteristic in Table 1)	*C. albicans*	3.13 mg/mL	Not studied	[53]
Brazilian green propolis –ethanolic extracts (chemical characteristic in Table 1)	*C. albicans*	3.13 mg/mL	Not studied	
Central European propolis –ethanolic extracts	*C. albicans*	6.25 mg/mL	Not studied	
Brazilian propolis	*C. albicans*—88 clinical isolates	Not studied	83.75–335	[72]
Brazilian propolis. Propolis extractive solution using ethanol	*C. albicans* ATCC90028 *C. albicans*—29 clinical isolated strains	68.35–546.87	Not studied	[69]
Cameroonian propolis, methanol–dichloromethane extract (three extracts)	*C. albicans* NR-29450	125–500	Not studied	[18]
	*C. krusei* ATCC 6258	250	Not studied	
	*C. parapsilosis* ATCC 22019	>500	Not studied	
	*C. glabrata*—clinically isolated strain	MIC > 500–250 µg/mL	Not studied	
Czech propolis (chemical characteristic in Table 1)	*C. albicans* (ATCC 90028)	0.6	1.2	[21]
	*C. albicans*—clinical isolated, No. 105366	1.2	2.5	
	*C. glabrata* (MYA 2950)	0.6	0.6	
	*C. glabrata*—clinical isolated, No. 105410	2.5	5	
	*C. glabrata*—clinical isolated No. 105413	0.6	1.2	
	*C. parapsisolis* (ATCC 22019)	0.6	0.6	
	*C. parapsisolis*—clinical isolated, No. 105328	2.5	2.5	
	*C. tropicalis* (ATCC 9968)	0.6	1.2	
	*C. krusei* (ATCC 90878)	1.2	2.5	
French poplar type propolis; 95% ethanolic extract	*C. albicans*	31.25	Not studied	[56]
	*C. glabrata*	MIC_80_ = 15.63	Not studied	
	*Aspergillus fumigatus*	250	Not studied	
French poplar type propolis 70% ethanolic extract	*C. albicans*	31.25	Not studied	[56]
	*C. glabrata*	MIC_80_ = 31.25	Not studied	
	*Aspergillus fumigatus*	250	Not studied	
French poplar type propolis –methanolic extract	*C. albicans*	31.25	Not studied	[56]
	*C. glabrata*	MIC_80_ = 31.25	Not studied	
	*Aspergillus fumigatus*	250	Not studied	
French poplar type propolis –dichloromethane extract (chemical characteristic in Table 1)	*C. albicans*	31.25	Not studied	[56]
	*C. glabrata*	MIC_80_ = 31.25	Not studied	
	*Aspergillus fumigatus*	250	Not studied	
French poplar-type propolis –water extract	*C. albicans*	>250	Not studied	[56]
	*C. glabrata*	MIC_80_ > 250	Not studied	
	*Aspergillus fumigatus*	250	Not studied	
German propolis (chemical characteristic in Table 1)	*C. albicans* (ATCC 90028)	5	>5	[21]
	*C. albicans*—clinical isolated, No. 105366	5	>5	
	*C. glabrata* (MYA 2950)	5	>5	
	*C. glabrata*—clinical isolated, No. 105410	>5	>5	
	*C. glabrata*—clinical isolated No. 105413	>5	>5	
	*C. parapsisolis* (ATCC 22019)	1.2	>5	
	*C. parapsisolis*—clinical isolated, No. 105328	>5	>5	
	*C. tropicalis* (ATCC 9968)	5	>5	
	*C. krusei* (ATCC 90878)	>5	>5	
Iranian propolis, 96% ethanolic extract	*C. albicans* ATCC10231	MIC_50_ = 21	MFC = 65	[63]
	*C. albicans* ATCC10231	MIC_90_ = 39	MFC = 65	
Iranian propolis, 50% ethanolic extract	*C. albicans* (23 clinical samples)	2.74 mg/mL	Not studied	[62]
Iranian propolis, 25% ethanolic extract	*C. albicans* (23 clinical samples)	9.01 mg/mL	Not studied	[58]
Irish propolis (chemical characteristic in Table 1)	*C. albicans* (ATCC 90028)	0.6	0.6	[21]
	*C. albicans*—clinical isolated, No. 105366	0.3	0.3	
	*C. glabrata* (MYA 2950)	0.3	0.6	
	*C. glabrata*—clinical isolated, No. 105410	0.6	0.1	
	*C. glabrata*—clinical isolated No. 105413	0.1	0.6	
	*C. parapsisolis* (ATCC 22019)	0.3	0.6	
	*C. parapsisolis*—clinical isolated, No. 105328	0.6	0.6	
	*C. tropicalis* (ATCC 9968)	0.2	0.3	
	*C. krusei* (ATCC 90878)	0.6	>0.6	
Nepalense propolis (chemical characteristic in Table 1)	*C. albicans* (ATCC 10231)	256	Not studied	[50]
Polish propolis, 50 samples collected from different regions of northern Poland	*C. albicans* (reference strains)	No information	0.31– >2.5%	[57]
	*C. albicans* (clinical isolates)	No information	0.08– >2.5%	
Polish propolis from southern Poland, 70% ethanolic extracts (5 extracts) (chemical characteristic in Table 1)	*Alternaria solani* ATCC 16022	2–8 mg/mL	4–8 mg/mL	[43]
	*Aspergillus niger* ATCC 9142	4–32 mg/mL	8–32 mg/mL	
	*Aspergillus ochraceus* KKP 124	8–16 mg/mL	8–16 mg/mL	
	*Botrytis cinerea* IOR 2110,	4–8 mg/mL	4–8 mg/mL	
	*Candida albicans* ATCC 10231	2–32 mg/mL	4–32 mg/mL	
	*Candida krusei* ATCC 14243	4–8 mg/mL	8–32 mg/mL	
	*Cladosporium cladosporioides* ATCC 16022	4–8 mg/mL	8–16 mg/mL	
	*Colletotrichum gloeosporioides* 62146	2–4 mg/mL	2–8 mg/mL	
	*Fusarium solani* ATCC 36031	2–4 mg/mL	4–16 mg/mL	
	*Mucor mucedo* ATCC 38694	4–8 mg/mL	4–8 mg/mL	
	*Penicillium expansum* KKP 774	8 mg/mL	16 mg/mL	
	*Penicillium chrysogenum* ATCC 10136	8–16 mg/mL	16–32 mg/mL	
	*Rhodotorula mucilaginosa* ATCC 66034	4–8 mg/mL	8–16 mg/mL	
	*Rhizopus stolonifer* ATCC 14037	4–8 mg/mL	32 mg/mL	
	*Saccharomyces cerevisiae* ATCC 9763	4–16 mg/mL	8–32 mg/mL	
Polish propolis; 70% ethanolic extract (chemical characteristic in Table 1)	*Aspergillus niger* ATCC 6275	7.5 mg/mL	No studied	[9]
	*Aspergillus versicolor* ATCC 11730	2.0 mg/mL	Not studied	
	*Aureobasidium pullulans* ATCC 9348	0.5 mg/mL	Not studied	
	*Paecilomyces variotii* ATCC 18502	5.0 mg/mL	Not studied	
	*Penicillium funiculosum* ATCC 11797	5.0 mg/mL	Not studied	
	*Penicillium cyclopium*	1.0 mg/mL	Not studied	
	*Trichoderma virens*	5.0 mg/mL	Not studied	
Polish propolis; 96% ethanolic extract (chemical characteristic in Table 1)	*Aspergillus niger* ATCC 6275	7.5 mg/mL	Not studied	[9]
	*Aspergillus versicolor* ATCC 11730	2.0 mg/mL	Not studied	
	*Aureobasidium pullulans* ATCC 9348	1.0 mg/mL	Not studied	
	*Paecilomyces variotii* ATCC 18502	7.5 mg/mL	Not studied	
	*Penicillium funiculosum* ATCC 11797	5.0 mg/mL	Not studied	
	*Penicillium cyclopium*	1.5 mg/mL	Not studied	
	*Trichoderma virens*	5.0 mg/mL	Not studied	
Polish propolis, dichloromethane extract	*C. albicans*	1.4 mg/mL	Not studied	[58]
	*C. glabrata*	1.25 mg/mL	Not studied	
	*C. tropicalis*	1.28 mg/mL	Not studied	
Polish propolis, methanolic extract	*C. tropicalis*	not active	Not studied	
	*C. albicans*	not active	Not studied	
	*C. glabrata*	not active	Not studied	
Polish propolis from Silesia region, ethanolic extracts	*C. albicans* (20 clinical isolates)	no information	0.312–5 [% *v*/*v*]	[60]
	*C. glabrata* (14 clinical isolates)	no information	to 5 [% *v*/*v*]	
	*C. krusei* (10 clinical isolates)	no information	not active	
Portuguese propolis, 80% ethanolic extract of materials collected from in north-eastern Portugal	*C. albicans*	MIC_50_ = 32.0 ± 3.2	Not studied	[64]
	*Aspergillus fumigatus*	MIC_50_ > 64.0	Not studied	
	*Trichophyton rubrum*	MIC_50_ = 14.5 ± 1.2	Not studied	
Portuguese propolis, 80% ethanolic extract of materials collected from the centre of Portugal	*C. albicans*	MIC_50_ = 43.1 ± 4.1	Not studied	[64]
	*Aspergillus fumigatus*	MIC_50_ > 64.0	Not studied	
	*Trichophyton rubrum*	MIC_50_ = 11.0 ± 0.9	Not studied	
Portuguese propolis, 80% ethanolic extract of materials collected from male hybrid species of *Populus*	*C. albicans*	MIC_50_ = 33.6 ± 3.5	Not studied	[64]
	*Aspergillus fumigatus*	MIC_50_ > 64.0	Not studied	
	*Trichophyton rubrum*	MIC_50_ = 38.9 ± 2.3	Not studied	
Portuguese propolis, 80% ethanolic extract of materials collected from female hybrid species of *Populus*	*C. albicans*	MIC_50_ = > 64.0	Not studied	[64]
	*Aspergillus fumigatus*	MIC_50_ > 64.0	Not studied	
	*Trichophyton rubrum*	MIC_50_ = 24.2 ± 2.1	Not studied	
Portuguese propolis, 80% ethanolic extact of materials collected from *Cistus ladanifer*	*C. albicans*	MIC_50_ = > 64.0	Not studied	[64]
	*Aspergillus fumigatus*	MIC_50_ > 64.0	Not studied	
	*Trichophyton rubrum*	MIC_50_ = 20.8 ± 1.8	Not studied	
Spanish propolis 70% ethanolic extract	*C. glabrata*—12 clinical isolated strains	60–240	MFC_50_ = 240	[16]
		MIC_50_ = 120	MFC_90_ = 480	
		MIC_90_ = 120		
